# Vitamin D Supplementation Guidelines for General Population and Groups at Risk of Vitamin D Deficiency in Poland—Recommendations of the Polish Society of Pediatric Endocrinology and Diabetes and the Expert Panel With Participation of National Specialist Consultants and Representatives of Scientific Societies—2018 Update

**DOI:** 10.3389/fendo.2018.00246

**Published:** 2018-05-31

**Authors:** Agnieszka Rusińska, Paweł Płudowski, Mieczysław Walczak, Maria K. Borszewska-Kornacka, Artur Bossowski, Danuta Chlebna-Sokół, Justyna Czech-Kowalska, Anna Dobrzańska, Edward Franek, Ewa Helwich, Teresa Jackowska, Maria A. Kalina, Jerzy Konstantynowicz, Janusz Książyk, Andrzej Lewiński, Jacek Łukaszkiewicz, Ewa Marcinowska-Suchowierska, Artur Mazur, Izabela Michałus, Jarosław Peregud-Pogorzelski, Hanna Romanowska, Marek Ruchała, Piotr Socha, Mieczysław Szalecki, Mirosław Wielgoś, Danuta Zwolińska, Arkadiusz Zygmunt

**Affiliations:** ^1^Department of Paediatric Propedeutics and Bone Metabolic Diseases, Medical University of Lodz, Lodz, Poland; ^2^Department of Biochemistry, Radioimmunology and Experimental Medicine, The Children’s Memorial Health Institute, Warsaw, Poland; ^3^Department of Pediatrics, Endocrinology, Diabetology, Metabolic Diseases and Cardiology of the Developmental Age, Pomeranian Medical University, Szczecin, Poland; ^4^Neonatal and Intensive Care Department, Medical University of Warsaw, Warsaw, Poland; ^5^Department of Pediatrics, Endocrinology and Diabetology with Cardiology Divisions, Medical University of Bialystok, Bialystok, Poland; ^6^Department of Neonatology and Neonatal Intensive Care Unit, The Children’s Memorial Health Institute, Warsaw, Poland; ^7^Mossakowski Medical Research Center, Polish Academy of Sciences, Department of Internal Diseases, Endocrinology and Diabetology, Central Hospital MSWiA, Warsaw, Poland; ^8^Department of Neonatology, Institute of Mother and Child, Warsaw, Poland; ^9^Department of Pediatrics, The Medical Centre of Postgraduate Education, Warsaw, Poland; ^10^Division of Clinical Genetics, Department of Molecular Biology and Genetics, School of Medicine in Katowice, Medical University of Silesia, Katowice, Poland; ^11^Department of Pediatric Rheumatology, Immunology, and Metabolic Bone Diseases, Medical University of Bialystok, Bialystok, Poland; ^12^Department of Pediatrics, Nutrition and Metabolic Diseases, The Children’s Memorial Health Institute, Warsaw, Poland; ^13^Department of Endocrinology and Metabolic Diseases, Medical University of Lodz, Polish Mother’s Memorial Hospital – Research Institute, Lodz, Poland; ^14^Faculty of Pharmacy with Laboratory Medicine, Medical University of Warsaw, Warsaw, Poland; ^15^Department of Geriatrics, The Centre of Postgraduate Medical Education, Warsaw, Poland; ^16^2nd Department of Pediatrics, Endocrinology, Diabetology, University of Rzeszow, Rzeszow, Poland; ^17^Department of Pediatrics and Pediatric Oncology, Pomeranian Medical University, Szczecin, Poland; ^18^Department of Endocrinology, Metabolism and Internal Diseases, Poznan University of Medical Sciences, Poznan, Poland; ^19^Department of Gastroenterology, Hepatology, Nutritional Disorders and Pediatrics, The Children’s Memorial Health Institute, Warsaw, Poland; ^20^Clinic of Endocrinology and Diabetology, The Children’s Memorial Health Institute, Warsaw, Poland; ^21^Faculty of Medicine and Health Sciences, Jan Kochanowski Univeristy, Kielce, Poland; ^22^1st Department of Obstetrics and Gynecology, Medical University of Warsaw, Warsaw, Poland; ^23^Department of Pediatric Nephrology, Wroclaw Medical University, Wroclaw, Poland

**Keywords:** vitamin D, vitamin D deficiency, recommendations of the experts, supplementation, treatment, vitamin D in Poland

## Abstract

**Introduction:**

Vitamin D deficiency is an important public health problem worldwide. Vitamin D deficiency confers a significant risk for both skeletal and non-skeletal disorders and a number of lifelong negative health outcomes. The objectives of this evidence-based guidelines document are to provide health care professionals in Poland, an updated recommendation for the prevention, diagnosis and treatment of vitamin D deficiency.

**Methods:**

A systematic literature search examining the prevention and treatment strategies for vitamin D deficiency was conducted. Updated recommendations were developed using the Grading of Recommendations, Assessment, Development and Evaluation system describing the strength of the recommendation and the quality of supporting evidence. Twenty-seven contributors representing different areas of expertise and medical specialties, including pediatricians, geriatricians, endocrinologists, epidemiologists, nephrologists, gynecologists and obstetricians evaluated the available published evidence related to vitamin D, formulated the goals of this document and developed a common consolidated position. The consensus group, representing six national specialist consultants and eight Polish and international scientific organizations/societies, participated in the process of grading evidence and drawing up the general and specific recommendations.

**Results:**

The updated recommendations define the diagnostic criteria for the evaluation of vitamin D status and describe the prevention and treatment strategies of vitamin D deficiency in the general population and in groups at increased risk of the deficiency. Age- and weight-specific recommendations for prevention, supplementation and treatment of vitamin D deficiency are presented, and detailed practice guidance is discussed regarding the management in primary and specialized health care.

**Conclusion:**

Vitamin D deficiency remains still highly prevalent in Poland, in all age groups. Currently, there is a great necessity to implement a regular supplementation with recommended doses and to develop an effective strategy to alleviate vitamin D deficiency in the population. These updated recommendations are addressed to health professionals and the authorities pursuing comprehensive health policies and should also be included in public health programs aimed at preventing a broad spectrum of chronic diseases.

## Introduction

Calcitriol [1,25-dihydroxycholecalciferol (1,25(OH)_2_D)], an active (hormonal) form of vitamin D, due to its action belongs to a broad group of hormones, being transcription factors of genes for target proteins. In contrast to other hormones of this group (e.g., androgens, estrogens, glucocorticosteroids, mineralocorticosteroids and progesterone), the synthesis of calcitriol is limited by availability of the substrate, 25-hydroxyvitamin D (25(OH)D). 25(OH)D is the most abundant metabolite of vitamin D and its serum concentration defines the status of vitamin D supply. Thus, vitamin D is a prohormone, and the term “vitamin D” should be referred both to ergocalciferol (vitamin D_2_) and cholecalciferol (vitamin D_3_), as products of conversion of ergosterol and 7-dehydrocholesterol (7-DHC). Biological action of calcitriol is mediated by the intracellular, highly specific vitamin D receptor (VDR)—a transcription factor modulated by a ligand that belongs to the family of genomic receptors for steroids, thyroid hormones and retinoids.

Available data indicate that vitamin D deficiency is a problem affecting general population and patients that is prevalent irrespective of latitude of residence, age, sex and race (1–3). In Poland, vitamin D deficiency of varying severity has been found in 90% of adults, children and adolescents (4–7). Vitamin D deficiency may be associated with its well-known calcemic effect as well as a broad spectrum of pleiotropic effects, the latter having been studied intensely in recent decades. Hence, the problem of vitamin D deficiency and its adequate supply represent an important issue in public health and clinical practice. Guidelines for vitamin D supplementation undergo modifications every few years, in view of new findings resulting in changing the paradigms. The global consensus on prevention and management of nutritional rickets was published in 2016 (8), which due to discrepancies with recommendations for the Central Europe (9) and previous Polish guidelines (10), evoked polemical discussion and dilemmas among doctors of many specialties, particularly among pediatric endocrine and diabetes specialists. Therefore, in 2017 The Board of the Polish Society of Pediatric Endocrinology and Diabetes came up with an initiative on verifying and updating ruling recommendations on prevention and management of vitamin D deficiency, both in the general population and in the risk groups. In cooperation with the European Vitamin D Association (EVIDAS) and other scientific societies and National Consultants, the Expert Panel was constituted to elaborate current guidelines for supplementation and treatment with vitamin D, based on recent literature reviews, personal clinical experience and critical discussion.

## Methods

The Expert Panel with the participation of National Consultants and Representatives of Scientific Societies, basing on the literature review and evaluation of strength and quality of evidence, developed current recommendations for prevention and treatment of vitamin D deficiency in the general population and in the risk groups.

For each point listed below, recommendations and a level of evidence are described, with following modification in the grading evidence: 1 = strong recommendation (application in the general population and in all patients in most circumstances, benefits clearly overweigh the risk) and 2-weak recommendation (consensus opinion of working group or to be considered; the best action may depend on circumstances, benefits and risk closely balanced or uncertain). Quality of evidence was assigned as follows: ⊕⊕⊕ high quality [prospective cohort or randomized controlled trials (RCT) studies, at low risk of bias]; ⊕⊕ moderate quality (observational or clinical trials with methodological flaws, inconsistent or indirect evidence) and ⊕ low quality (case reports, case series or non-systematic clinical observations). The Expert Panel has confidence that vitamin D supplementation or vitamin D deficiency treatment are, on average, safe and beneficial when used according to the strong recommendations. Weak recommendations necessitate more personalized consideration, and, on average, are safe and beneficial as well.

## Recommendations—2018 Update

### General Recommendations

(1) Prophylactic dosing of vitamin D in the general population should be individualized depending on age, body weight, insolation (season, time of year), sun exposure of an individual, dietary habits and lifestyle (1⊕⊕);
(2) Prophylactic dosing of vitamin D in the risk groups of vitamin D deficiency (Table 1) should be implemented according to arrangements for the general population; if no specific practice guidelines are established, the maximal admissible doses for a given age group in the general population are recommended for use in the risk groups of vitamin D deficiency (2⊕⊕);
(3) In the general population, in case of vitamin D deficiency ascertained by laboratory assays, the administration of vitamin D should be based on doses dependent on serum 25(OH)D concentration and chronological (calendar) age, in relation to body weight (2⊕⊕);
(4) In the risk groups, the dosing of vitamin D in case of vitamin D deficiency ascertained by laboratory assays, should be based on doses dependent on the 25(OH)D concentration and age, with regard to the nature of the disease, medical therapy and body weight (1⊕⊕);
(5) In the general population, the specific indications for 25(OH)D assay testing are not established and 25(OH)D concentration screening is not recommended (1⊕⊕);
(6) In the risk groups, the evaluation of vitamin D status, based on 25(OH)D concentration assay, is recommended (1⊕⊕);

**Table 1:** Indications for assessment of 25(OH)D concentration in serum—groups at risk of vitamin D deficiency.

Disorders	Examples of diagnoses
Disorders of the locomotor system	Rickets, osteomalacia, osteoporosis, bone pains, bone deformations, postural defects, recurrent low energy fractures and aseptic osteonecrosis

Disorders of calcium-phosphorus metabolism	Disorders of calcemia, calciuria, phosphatemia, phosphaturia, hypophosphatasia and hiperphosphatasia

Chronic treatment with some medications	Chronic corticosteridotherapy, treatment with ketoconazole, antiretroviral and antiepileptic therapy

Maldigestion and malabsorption	Maldigestion and malabsorption syndromes, cystic fibrosis and chronic inflammatory bowel disease

Liver diseases	Liver failure, cholestasis, posttrasplant state and non-alcoholic fatty liver disease (NAFLD)

Kidney diseases	Renal failure, posttransplant state and nephrocalcinosis

Endocrine disorders	Hyper- and hypoparathyroidism, hyper- and hypothyroidism, diabetes type 1, growth hormone deficiency, anorexia nervosa and autoimmune polyglandular syndromes

Disorders of somatic development	Short stature, tall stature, obesity and cachexia

Developmental delay	Delay of psychomotor development and intellectual disability

Diseases of the nervous system	Cerebral palsy, chronic immobilization, autism, multiple sclerosis, epilepsy, seizures of unknown etiology, miopathy and muscular dystrophy

Allergy	asthma, atopic dermatitis

Autoimmune diseases	Collagen diseases, rheumathoid arthritis, autoimmune diseases of the skin, diabetes type 1 and Hashimoto disease

Immune disorders	Recurrent infections of the respiratory tract, asthma, recurrent and chronic inflammatory states of other systems

Neoplasms	Blood cancer, malignancy of the lymphatic system and other organs, tumors and states after oncologic treatment

Cardiovascular diseases	Arterial hypertension and ischemic heart disease

Metabolic diseases	Diabetes type 2, lipid disorders, obesity and metabolic syndrome

### Recommendations for Vitamin D Supplementation in the General Population

#### Neonates Born at Term and Infants

- 0–6 months: 400 IU/day from first days of life, regardless the way of feeding (1⊕⊕⊕);
- 6–12 months: 400–600 IU/day, depending on daily amount of vitamin D taken with food (1⊕⊕⊕);

#### Children (1–10 Years)

- In healthy children sunbathing with uncovered forearms and legs for at least 15 min between 10.00 and 15.00 h, without sunscreen in the period from May to September, supplementation is not necessary, although still recommended and safe (1⊕⊕⊕);
- If above insolation guidelines are not fulfilled, supplementation of 600–1000 IU/day is recommended, based on body weight and the dietary vitamin D intake, throughout a year (1⊕⊕⊕);

#### Adolescents (11–18 Years)

- In healthy adolescents sunbathing with uncovered forearms and legs for at least 15 min between 10.00 and 15.00 h, without sunscreen in the period from May to September, supplementation is not necessary, although still recommended and safe (1⊕⊕⊕);
- If above insolation guidelines are not fulfilled, supplementation of 800–2000 IU/day is recommended, based on body weight and the dietary vitamin D intake, throughout a year (1⊕⊕⊕);

#### Adults (19–65 Years)

- In healthy adults sunbathing with uncovered forearms and legs for at least 15 min between 10.00 and 15.00 h, without sunscreen in the period from May to September, supplementation is not necessary, although still recommended and safe (1⊕⊕⊕);
- If above insolation guidelines are not fulfilled, supplementation of 800–2000 IU/day is recommended, based on body weight and the dietary vitamin D intake, throughout a year (1⊕⊕⊕);

#### Seniors (>65–75 Years) and People With a Dark Complexion

- Due to decreased efficacy of the skin synthesis, supplementation of vitamin D in the dose of 800–2,000 IU/day, based on body weight and the dietary vitamin D intake is recommended throughout a year (1⊕⊕⊕);

#### Eldest Seniors (>75 Years)

- Due to decreased efficacy of the skin synthesis, potential malabsorption and altered metabolism of vitamin D, supplementation of 2,000–4,000 IU/day, based on body weight and the dietary vitamin D intake is recommended throughout a year (2⊕⊕);

#### Pregnant and Lactating Women

- Women planning pregnancy should receive adequate vitamin D supply, the same as in the general adult population, if it is possible under the control of 25(OH)D concentration (1⊕⊕⊕);
- When pregnancy is confirmed, supplementation should be carried out under the control of 25(OH)D concentration, to maintain optimal concentrations within ranges of >30–50 ng/ml (1⊕⊕⊕);
- If the assessment of 25(OH)D concentration is not possible, it is recommended to use vitamin D at a dose of 2,000 IU/day, throughout pregnancy and lactation (1⊕⊕⊕);

#### Preterm Neonates

##### Neonates Born at ≤32 Weeks of Gestation

- It is recommended to start supplementation at a dose of 800 IU/day from the first days of life (if enteral nutrition is possible), regardless the way of feeding (1⊕⊕⊕);
- Supplementation should be carried out under the control of 25(OH)D concentration, both during hospitalization (the first control after 4 weeks of supplementation), as well as in the out-patient care (1⊕⊕);
- When achieving a total dose of 1,000 IU/day, combining supplements and diet, there is a risk of vitamin D overdose, particularly in neonates with birth weight <1,000 g (1⊕⊕⊕);

##### Neonates Born at 33–36 Weeks of Gestation

- 400 IU/day from the first days of life, regardless the way of feeding (1⊕⊕⊕);
- There is no need to assay 25(OH)D concentrations routinely (1⊕⊕⊕);
- Supplementation under the control of 25(H)D concentration should be considered in children in the risk groups (parenteral nutrition >2 weeks, ketoconazole >2 weeks, anticonvulsant treatment, cholestasis and birth weight <1,500 g) (2⊕⊕);

### Supplementation in Groups at Risk of Vitamin D Deficiency

- A special risk group comprises obese individuals who require double dose of vitamin D in regard to doses recommended for age-matched peers with normal body weight (1⊕⊕⊕); obesity in children and adolescents is defined as BMI > 90th percentile for age and gender; obesity in adults and the elderly is defined as BMI 30+ kg/m^2^;
- In groups at risk of vitamin D deficiency (Table 1), supplementation should be implemented and followed up under the control of 25(OH)D concentrations, in order to maintain the optimal concentration of >30–50 ng/ml (2⊕⊕);
- If the assessment of 25(OH)D concentration is not possible, dosing should be carried out according to the guidelines for the general population at the maximal doses for a given age group (2⊕⊕);

### Supplementation in Groups at Risk of Vitamin D Hypersensitivity

- Prior to initiating the supplementation, the probability of vitamin D hypersensitivity should be assessed if feasible (hypercalcemia, hypercalciuria, nephrocalcinosis, nephrolithiasis, *CYP24A1* gene mutation, *SLC34A1* gene mutation or history of other types of vitamin D hypersensitivity in an individual or family members). This recommendation applies to all age groups as well as to groups at the risk of vitamin D deficiency (1⊕⊕⊕);
- In groups at the risk of vitamin D hypersensitivity, supplementation should be supervised and carried out carefully and in an individual manner, preferably under the control of calcium-phosphate variables, particularly calcemia, calciuria, parathormone (PTH), 25(OH)D and 1,25(OH)_2_D (1⊕⊕);

### Principles of Supplementation and Treatment With Vitamin D Based on 25(OH)D Concentrations

- A single loading dose of vitamin D is not recommended in Poland (2⊕⊕);
- Vitamin D dosing should be based on 25(OH)D concentrations and antecedent prophylactic management (2⊕⊕);
- The diagnostic standards include simultaneous assays of 25(OH)D_2_ and 25(OH)D_3_ [25(OH)D TOTAL], with intraassay variation <5% and interassay variation <10%, being subject to quality assurance by the certifying system DEQAS (2⊕⊕);

#### Toxic Concentration >100 ng/ml (1⊕⊕⊕)

- Vitamin D supplementation has to be stopped forthwith; calcemia and calciuria should be assessed, and 25(OH)D concentration should be monitored at 1-month intervals until 25(OH)D concentrations of ≤50 ng/ml are reached (1⊕⊕⊕);
- Vitamin D intoxication is defined as the state in which the 25(OH)D concentration >100 ng/ml is accompanied by hypercalcemia, hypercalciuria and apparent PTH suppression (1⊕⊕⊕);
- In case of clinical symptoms of vitamin D intoxication, treatment should be immediately initiated (1⊕⊕⊕);
- Verify if previously used supplementation was appropriate, and correct the management accordingly (regularity of intake, dosing, type of preparation and the way of supply) (2⊕⊕);
- There is a possibility to re-entry vitamin D supplementation at doses recommended for peers from the general population, after reaching normocalcemia, normocalciuria and 25(OH)D concentrations ≤50 ng/ml, followed by excluding vitamin D hypersensitivity (2⊕⊕);

#### High Concentrations >50–100 ng/ml (1⊕⊕⊕)

- Verify if previously used supplementation was appropriate, and correct the management accordingly (regularity of intake, dosing, type of preparation and the way of supply) (2⊕⊕);

##### Concentrations >75–100 ng/ml (2⊕⊕)

- Vitamin D intake should be suspended for 1–2 months (2⊕⊕);
- In neonates, infants and toddlers, calcemia and calciuria should be assessed, vitamin D hypersensitivity should be excluded and the control assay of 25(OH)D concentration should be carried out (2⊕⊕);
- There is a possibility to re-entry vitamin D supplementation at minimal doses recommended for peers from the general population, after 1–2 months or, in case of neonates, infants and toddlers after reaching 25(OH)D concentrations ≤50 ng/ml (2⊕⊕);

##### Concentrations >50–75 ng/ml (2⊕⊕)

- If vitamin D supplementation was appropriate, it is recommended to reduce the dose by 50%, and to consider assessment of 25(OH)D concentration within the consecutive 3-month period (2⊕⊕);
- If vitamin D was supplemented at doses higher than recommended, the vitamin D supply should be ceased for 1 month, and then doses recommended for peers from the general population should be started (2⊕⊕);

#### Optimal Concentration >30–50 ng/ml (1⊕⊕⊕)

- Continue previous management (1⊕⊕⊕);

#### Suboptimal Concentration >20–30 ng/ml (1⊕⊕⊕)

- Verify if previously used supplementation was appropriate, and correct the management accordingly (regularity of intake, dosing, type of preparation and the way of supply) (2⊕⊕);
- If vitamin D supplementation was appropriate, it is recommended to increase the dose by 50% and to consider the assessment of 25(OH)D concentration in 6-month time (2⊕⊕);
- If vitamin D was not supplemented previously, it is recommended to start vitamin D intake at doses recommended for peers from the general population (2⊕⊕);

#### Deficiency >10–20 ng/ml (1⊕⊕⊕)

- Verify if previously used supplementation was appropriate, and correct the management accordingly (regularity of intake, dosing, type of preparation and the way of supply) (2⊕⊕);
- If vitamin D supplementation was appropriate, it is recommended to increase the dose by 100% and to assess 25(OH)D concentration in 3-month time (2⊕⊕);
- If vitamin D was not supplemented previously, it is recommended to start vitamin D intake at maximal doses recommended for peers from the general population and to assess 25(OH)D concentration in 3-month time (2⊕⊕);
- In patients with skeletal symptoms (bone deformations, bone pain, history of fragility fractures), it is indicated to assess calcium-phosphate metabolism [Ca, PO_4_, alkaline phosphatase activity (ALPL), PTH, Ca/creatinine ratio in urine], and, if available—bone mineral density using dual-energy X-ray absorptiometry (DXA) (2⊕⊕);

#### Severe Deficiency 0–10 ng/ml (1⊕⊕⊕)

- Verify if previously used supplementation was appropriate, and correct the management accordingly (regularity of intake, dosing, type of preparation and the way of supply) (2⊕⊕);
- Therapeutic doses should be implemented, based on age and body weight; the repeated control assay of 25(OH)D concentration should be performed after 1–3 months of therapy (1⊕⊕⊕);
  - From birth to 12 months of age: 2,000 IU/day (1⊕⊕⊕);
  - 1–10 years: 3,000–6,000 IU/day (1⊕⊕⊕);
  - >10 years: 6,000 IU/day (1⊕⊕⊕);
- Treatment of severe deficiency should be carried out for 3 months or until the 25(OH)D concentration of >30–50 ng/ml is reached, then it is recommended to use consecutive maintenance dose, i.e., a prophylactic dose recommended for general population, in relation to age and body weight (1⊕⊕⊕);
- In patients with skeletal symptoms and bone mineral disorders (bone deformations, bone pain, history of fragility fractures), it is necessary to assess and monitor parameters of calcium-phosphate metabolism (Ca, PO_4_, ALPL, PTH and Ca/creatinine ratio in urine), and if available—to examine bone mineral density using DXA (2⊕⊕);

### Principles of Calcium Intake During Supplementation and Treatment With Vitamin D

- During supplementation and treatment with vitamin D, an appropriate dietary calcium intake should be assured (Table 2) (2⊕⊕);
- If adequate dietary calcium intake is not possible, an additional pharmacological supplementation with calcium salts preparations is recommended, preferably in divided doses, which should be taken with meals (2⊕⊕);

**Table 2:** Sources of calcium in the diet, equivalent to one glass/one serving of milk (240 mg calcium).

Basic source of calcium	Equivalents
1 average glass of milk = 240 mg of calcium	1 small mug of yogurt (150 g)
	1 glass of kefir
	1 glass of buttermilk
	35 dag of curd cheese
	2 small triangles of processed cheese
	2 slices of cheese
	2 packages of cottage cheese
	100 g sardines
	100 g almonds
	130 g hazelnuts
	150 g beans (dry seeds)
	260 g spinach
	350 g cabbage

## Evidence base for Updated Polish Recommendations

### Sources of Vitamin D

#### Skin Synthesis of Vitamin D

Most of vitamin D in humans is produced in the skin, in the keratinocytes of the epidermal germinative layer from 7-DHC, after exposure to sunlight radiation of wavelength of 280–315 mm [ultraviolet radiation B (UVB)]. Under the influence of the absorbed energy, 7-DHC undergoes transformation to pre-vitamin D_3_, and then, due to a thermoconversion, to vitamin D_3_. The latter enters the bloodstream, where it is bound to vitamin D-binding protein (DBP). It is estimated that skin synthesis may cover 80–100% of vitamin D daily requirement. In Poland, the skin synthesis of vitamin D may be effective in spring and summer only (from May to September), between 10:00 and 15:00, i.e., at the season and time of a day providing an appropriate angle of sunlight, air temperature favoring sunbath, and predominant cloudless weather (12, 13). In such conditions, an exposure of at least 18% of the body surface (i.e., uncovered forearms and lower limbs) for approximately 15 min should constitute half of minimal erythemal dose (MED; 1 MED results in a light pinkness of the skin) and may lead to natural synthesis of vitamin D in a quantity equivalent to 2,000–4,000 IU/day (12–14). Consequently, the exposure of nearly 100% of the body surface of an adult person may yield 10,000 IU/day. So far no reports showing the risk of obtaining toxic quantities of vitamin D after excessive exposure to sunlight have been published (at least in healthy subjects). This is explained by the fact that possible excess of vitamin D and previtamin D (immediate precursor in the cholesterol biosynthetic pathway) are photodegraded (isomerized) into inactive metabolites—tachysterol, lumisterol, suprasterols and 5,6-trans-vitamin D_3_. In the period from October to March, in the regions above 35° north latitude (including Poland; 49°N–54°N), the skin synthesis is considered as not effective (12–15).

Intrinsic and environmental factors such as cloud cover, air pollution, intensive skin pigmentation, advanced age, excessive usage of sun protection cosmetics with sun protection factor above 15, significantly extend the exposure time necessary for achieving sufficient vitamin D supply. The above factors may totally prevent vitamin D skin synthesis even if the appropriate amount of time spent in the sunlight during spring and summer is provided. There are also individuals not adherent to the practice guidelines, or those who fail to follow recommended sensible exposure to sunlight due to specific contraindications. Sun exposure tends to be very limited among children and adolescents because of their indoor extra activities, and also in a large proportion of adult population due to the type of job. In neonates, infants and children younger than 3 years of age, the direct exposure to sunlight without sunscreen is not recommended (http://pediatrics.aappublications.org/content/pediatrics/early/2011/02/28/peds.2010-3501.full.pdf). Other reasons may include chronic diseases preventing from outdoor activities, cancer phobia or fear of skin aging (6, 14, 15).

#### Diet

The diet is the alternative source of vitamin D for humans; however in natural conditions, at least in Poland, it is a significantly less effective source, compared to skin synthesis. It is estimated that balanced diet covers up to 20% of the required daily vitamin D intake. Dietary vitamin D is present in two forms. In the food products of animal origin the dominant form of vitamin D is cholecalciferol (vitamin D_3_), and that of plant and fungal origins—ergocalciferol (vitamin D_2_). Natural sources of vitamin D mainly comprise fish, such as eel, wild salmon, herring and to a lesser extent—egg yolk, cheese, milk and some mushrooms (Table 3). Evaluation of nutritional habits and food composition in different populations showed that, when an additional source such as skin synthesis is scarce, even varied and balanced diet cannot be regarded to match the complete vitamin D requirement. Therefore, an appropriate vitamin D supplementation plays a crucial role in maintenance of the optimal health outcomes (3, 5, 16).

**Table 3:** Vitamin D content in selected nutritional products in Poland (9, 11).

Product	Vitamin D content (40 IU = 1 µg)
Fresh eel	1,200 IU/100 g
Fresh wild salmon	600–1,000 IU/100 g
Herring in oil	808 IU/100 g
Marinated herring	480 IU/100 g
Salmon (cooked/baked)	540 IU/100 g
Fresh farmed salmon	100–250 IU/100 g
Canned fish (tuna, sardines)	200 IU/100 g
Mackerel (cooked/baked)	152 IU/100 g
Fresh codfish	40 IU/100 g
Shiitake mushrooms	100 IU/100 g
Egg yolk	54 IU/egg yolk
Cheese	7.6–28 IU/100 g
Human milk	1.5–8 IU/100 ml
Human milk during vitamin D supplementation	~20 IU/100 ml
Cow’s milk	0.4–1.2 IU/100 ml
First infant formula (0–6 months)	40–60 IU/100 ml
Follow-on formula (7–12 months)	56–76 IU/100 ml
Growing-up formula (2–3 years)	70–80 IU/100 ml

In terms of prevention of vitamin D deficiency at the population level, a mandatory fortification of selected food products (milk, dairy products, cereals, orange juice, margarine and pasta) is provided in some countries (17). The extent of fortification varies across different world regions, depending on health policy and governmental strategies. So far, the food fortification has not been customarily applied globally or locally in Poland, resulting in pandemic of vitamin D deficiency according to some reports (1–3). Milk formulas for infants and toddlers are an exception, as these products are enriched with vitamin D in a standard way (Table 3). The amount of 1 l of commercial formula milk is proven to cover the daily vitamin D requirement sufficiently, at least in context of prevention of vitamin D deficiency and nutritional rickets. It should be underlined that the vitamin D supplementation is necessary in breast-fed infants. The vitamin D content in human milk is fluctuating and small (about 40 IU/l), and seems insufficient for a growing child, even if standard recommended intakes of vitamin D are reassured in a breast-feeding mother (18, 19).

#### Pharmacological Preparations of Vitamin D

Cholecalciferol (D_3_) is the most common preparation used as supplementation and treatment of vitamin D deficiency in Poland and Europe, unlike in the USA, where ergocalciferol (D_2_) is largely used. In Poland, vitamin D_3_ is available over-the counter at daily doses of 400, 500, 800, 1,000, 2,000 and 4,000 IU. Vitamin D_3_ is also available as multivitamin preparations, in composite calcium supplements, cod liver oil and, less frequently, in some food products fortified with vitamin D. The administration of vitamin D as a combination containing calcium or vitamin K2 (MK7) or in conjunction is not recommended presently. Efficacy of simultaneous intake of the vitamins K2 and D, as a factor preventing calcification of vessels and soft tissues, as well as enhancing bone mineralization, has not been proven. Vitamin D should not be administered together with high-fiber cereals (oatmeal and bran), resins binding steroids (colestyramine), laxatives or stool softeners.

Calcifediol, 25(OH)D_3_, is an essential preparation improving vitamin D supply, however, this medication is used mainly in patients with impaired hepatic metabolism of vitamin D, coincident chronic liver diseases, cholestasis, long-term therapy with glucocorticosteroids and anticonvulsants (9).

Active metabolites and analogs of vitamin D, such as alfacalcidol (1αOHD_3_), calcitriol [1α,25(OH)_2_D_3_] and paricalcitol [19nor1α25(OH)_2_D_2_] should not be considered as an alternative way of vitamin D supplementation, and in consequence, monitoring of the therapy based on active metabolites and analogs, using serial prospective evaluation of 25(OH)D concentration demonstrates a limited value. Alfacalcidol is most often used in cases presenting with impaired renal metabolism of vitamin D and in diseases with a decreased activity of 1α-hydroxylase, such as renal failure, nephrotic syndrome, chronic kidney disease, hypophosphatemic rickets and other vitamin D-resistant rickets, as well as in hypoparathyroidism. Calcitriol has been assigned similar application; however, it is used less frequently and is less available in Poland. Paricalcitol is a newly developed vitamin D analog, used for prevention and treatment of secondary hyperparathyroidism resulting from chronic renal failure (20–22). It should be underlined again, that the use of analogs and active metabolites of vitamin D does not lead to expected changes of 25(OH)D concentrations and is not an alternative mode for supplementation with the use of vitamin D (or with calcifediol in specific cases). The use of analogs does not detract the patient from necessity to a concurrent vitamin D intake, just in the context of pleiotropic health benefits.

### Vitamin D Metabolism

Cholecalciferol—produced in the skin, and cholecalciferol (and ergocalciferol)—absorbed in the small intestine (from diet and pharmacological supplements), are transported to the liver and bound to the DBP. In the liver, the first stage of biosynthesis of an active vitamin D is activated. After enzymatic hydroxylation at the carbon-25, 25-hydroxyvitamin D—25(OH)D is formed. This reaction is catalyzed by 25-hydroxylase, which compromises a group of hydroxylases, being part of cytochrome P450 (CYP27A1, CYP3A4 and CYP2R1) (23, 24). The concentration of 25(OH)D in the serum is the best indicator of vitamin D status, mainly due to a higher stability and a longer half-time of 25(OH)D (2–3 weeks), as well as relatively high levels of concentration (expressed in ng/ml or nmol/l) in comparison to the 1,25(OH)_2_D, being under multifactoral regulation and at markedly lower concentrations (half-time 4–6 h, concentration expressed in pg/ml or pmol/l).

The 25(OH)D bound to DBP is subsequently transported from the liver to kidneys (as well as to many other tissues, organs and cells), where an active form of vitamin D—the 1α25(OH)_2_D is formed via the next key enzyme—1α-hydroxylase (CYP27B1). The activity of 1α-hydroxylase (CYP27B1) is regulated by many factors, including calcium concentration, PTH, fibroblast growth factor 23 (FGF-23) and Klotho, as well as by 1α,25(OH)_2_D itself, through the mechanism of a negative feedback loop (24, 25). Both active forms of vitamin D [1α,25(OH)_2_D_2_ and 1α,25(OH)_2_D_3_] are characterized by similar properties. Due to common occurrence in the nature and the availability of pharmaceutic preparations in Poland, vitamin D_3_—cholecalciferol is practically the only one in customary use. Therefore, the prevalent measurable form of an circulating active metabolite is represented by 1α,25-dihydroxycholecalciferol, i.e., calcitriol.

The concentrations of 25(OH)D and 1,25(OH)_2_D are tightly regulated by enzymatic cleavage in reaction of 24-hydroxylase (CYP24A1), which catalyzes the hydroxylation of calcidiol and calcitriol to metabolites of low biological activity, finally transformed to 24,25(OH)_2_D and calcitrionic acid, respectively. Calcitriol is a factor inducing 24-hydroxylase expression, present practically in all target cells which are under vitamin D action. This is a feedback system regulating concentration of active metabolites of vitamin and preventing hypervitaminosis D (25). The above metabolic pathway may be disturbed in case of impaired catabolism of 25(OH)D and 1,25(OH)_2_D (mutations of *CYP24A1* gene coding 24-hydroxylase) (25) or excessive synthesis of 1,25(OH)_2_D directly resulting from mutation of *SLC34A1* gene coding sodium-phosphate co-transporter (NaPi-IIA) in the kidney (26). In both cases, the risk of hypervitaminosis D is increased even if prophylactic doses of vitamin D are used.

### Calcemic Effects of Vitamin D

Classic and well recognized action of vitamin D consists of its key role, besides PTH and calcitonin, in regulation of calcium and phosphorus homeostasis. The main effector organs involved in the regulation of calcium and phosphorus homeostasis by vitamin D action include intestine, bones and kidney. In the intestine, under the influence of 1,25(OH)_2_D, synthesis of a protein binding calcium and calcium absorption are increased, in bones—calcium and phosphates (in case of hypocalcemia) are released, and in kidneys—calcium is reabsorbed with input from PTH activity. The main procalcemic action of calcitriol is the inhibition of PTH secretion in the feedback via calcitriol, resulting in the increase of calcium and phosphates concentrations in serum (27).

Vitamin D action on bone metabolism is mediated by the receptor activator of nuclear factor NF-κB (RANK)/RANK ligand (RANKL) system, responsible for osteoclastogenesis. Calcitriol increases RANKL expression in osteoblasts, which in turn activates RANK receptors in osteoclast precursors leading to formation of mature osteoclasts. The resorption action of osteoclasts releases calcium and phosphates from the skeletal system into circulation (28).

Vitamin D, due to its action regulating calcium-phosphorus metabolism, plays a significant role mainly in tissues and organs rich in these minerals, i.e., the skeletal system and teeth. Vitamin D deficiency in children is a classic risk factor for nutritional rickets, a disease presenting with bone deformations of varying severity as well as impaired mineralization and decreased bone mass. In adults and adolescents, after growth plate fusion, vitamin D deficiency may lead to osteomalacia and osteoporosis. In all age groups, a severe vitamin D deficiency is related to bone pain of various intensity and localization (predominantly in the lower limbs and feet) as well as increased susceptibility to bone fractures. Advanced stages of nutritional rickets and osteomalacia may be even the life-threatening conditions, characterized not only by bone deformations, but also by hypocalcemic seizures (29, 30), tetany (31, 32), severe bone pain and significant muscle weakness (33, 34), hypocalcemic cardiomiopathy, even resulting in circulatory failure (35–38), and by disorders of psychomotor and physical development (39), including short stature (4, 40).

In the scenario of vitamin D deficiency, a three-stage regulatory mechanism was described. Initially, a compensatory increase of PTH secretion to sustain normocalcemia is observed. However, PTH reveals an ability to auto-regulate, and a relative resistance to PTH may develop resulting in decreased calcium concentrations and increased phosphate concentrations (similarly to a PTH resistance in pseudohypoparathyroidism). At this stage, typical symptoms of hypocalcemia, including tetanic convulsions, may occur. Osteopenia is visible on radiographs, without typical rickets lesions. In the next stage, when the severity of vitamin D deficiency continues to progress and PTH secretion is further stimulated, a PTH resistance gets overcome leading to improved calcemia but also to hypophosphatemia and clinical and radiological manifestation of rickets. ALPL increases then, whereas concentration of 1,25(OH)_2_D is normal or increased. At the final stage, the vitamin D deficiency becomes very severe and 1,25(OH)_2_D synthesis is markedly inhibited, the calcitriol concentration decreases, and subsequently absorption of both calcium and phosphorus is impaired, along with persistent elevation of PTH and increased ALPL (18).

The mechanism described above highlights that results of biochemical assays may reveal varied values, depending on the severity of vitamin D deficiency. Therefore, isolated assays of 1,25(OH)_2_D concentrations may be misleading. In moderate vitamin D deficiency, as well as in the course of treatment, concentration of calcitriol may be normal or high, whereas in overdosage of vitamin D it may be normal or decreased (41, 42). These alternations prove a limited clinical utility of 1,25(OH)_2_D assays, as this metabolite shows low stability and is a subject to multifactorial influences, including hormonal regulation.

### Pleiotropic Action of Vitamin D

Development of molecular studies and discovery of VDR in many tissues, which do not take part in calcium-phosphorus metabolism, have initiated an era of intensive research on other non-classic, extra-skeletal functions of vitamin D (43–46). Furthermore, 1,25(OH)_2_D concentrations accessible in serum originate primarily from the kidneys and are connected with classic endocrine functions, however, enzymatic activity of extra-renal 1-alpha-hydroxylases that convert 25(OH)D to 1,25(OH)_2_D in numerous organs, tissues and cells enables important local autocrine and paracrine functions of this “extra-renal” calcitriol. Extra-renal vitamin D metabolic pathways (both anabolic and catabolic mechanisms) are regulated independently of the PTH- and FGF-23-mediated pathways. Importantly, the expression of both VDR and extra-renal 1-alpha-hydroxylases in almost all human tissues provides a sound scientific basis to postulate that vitamin D is important for overall human health.

The activity of vitamin D in effector tissues is exerted through its genomic and non-genomic action. An active form of vitamin D in a number of tissues and cells binds with VDR in the cell nucleus and then it forms a heterodimer with 9-cis retinoic acid receptor (RXR), which shows property of a transcription factor, so the genomic action is initiated. It is estimated that in this way calcitriol takes part in regulation of several hundred genes in the human genome (47, 48). Non-genomic effects are mediated by a receptor localized in a cellular membrane, which is different from the nuclear receptor, and it triggers intracellular metabolic pathways, modulating effects resulting from the gene expression (49, 50). The non-genomic, rapid responses related to calcitriol apply to the regulation of ion channels, phosphatases, phospholipases, kinases and signaling factors that may independently regulate gene expression and its products (49–52).

It has been reported that calcitriol takes part in numerous physiological processes, including enhancement of proliferation and differentiation of immune cells (52, 53). It induces apoptosis of neoplastic cells and inhibits their multiplication (52, 53), increases production of cathelicidin and beta-defensin (51, 54–56), modulates lymphocyte activity (51), Th1 and Th2 lymphocyte ratio (51, 57), decreases concentrations of proinflammatory cytokines (IL-1, TNFα), simultaneously increasing anti-inflammatory cytokines (IL-4, IL-5 and IL-10) (51, 58), decreases renin secretion and in this way reduces activity of the renin–angiotensin–aldosteron system (59, 60), inhibits angiogenesis (61–63), and has favorable effect on calcification processes in blood vessels (64–67), stimulates synthesis of neurotrophic factors (68, 69) and inhibits fibrosis in kidneys (70, 71). Vitamin D deficiency decreases insulin secretion (72). Vitamin D also shows strong immunomodulating action (58, 73). High content of VDR in cells of the immunocompetent system, particularly in macrophages, dendritic cells and lymphocytes T and B supports the concept of the essential role of vitamin D in anti-infectious immunity, in the course of acute and chronic inflammatory processes as well as in autoimmune diseases (45, 46, 58, 73).

The pleiotropic aspect of vitamin D action was tested in numerous observational studies, suggesting association between low serum concentration of 25(OH)D and increased risk of neoplasms (among others cancers of the colon, breast, ovary, prostate, pancreas, skin, kidneys, brain tumors, multiple myeloma and leukemia) (74–78), immunological diseases [multiple sclerosis (79–82), asthma (83, 84), non-specific inflammatory bowel diseases (85, 86) and systemic lupus erythematosus (87, 88)], autoimmune endocrine disorders [diabetes type 1 (89, 90), Addison disease (91, 92), Hashimoto disease (93, 94), Graves-Basedow disease (95, 96) and autoimmune polyendocrine syndromes (97)], immunodeficiencies and recurrent infections (98) (i.e., tuberculosis and influenza), components of metabolic syndrome (including arterial hypertension and cardiovascular diseases, atherosclerosis, ischemic heart disease, diabetes type 2 and obesity) (99–105), as well psychiatric disorders [depression (106), schizophrenia (107, 108)] and neurodegerative diseases [dementia (109–111), Alzheimer disease (112, 113), deterioration of cognitive functions (109, 110, 114)]. Vitamin D deficiency is also associated with increased mortality in the general population (115–117), in patients in intensive care units (118–120), and in patients with neoplasms (121). A better vitamin D supply, reflected by an optimal 25(OH)D concentration, may alleviate invasiveness of a neoplastic process and micrometastases, as well as improve disease prognosis, and also limit risk of recurrence (122). Largely conducted RCT studies, however, have not shown consistent results, likely due to a short time of follow-up, type of regimen and different levels of a supplementary dose (123, 124). Nevertheless, many of these studies showed health benefits related to vitamin D supplementation. Presently, however, it cannot be stated explicitly that vitamin D deficiency is a direct cause of many disorders and their sequelae, and the hypothesis of reverse causality gains significance in the light of systematic reviews of meta-analyses and RCTs (125, 126). Taking into account on-going studies presenting potential health benefits and negligible health risk resulting from supplementation and maintenance of optimal and safe 25(OH)D concentrations, it is indicated to recommend prophylactic administration of vitamin D in the general population, when skin synthesis is insufficient for different reasons.

### Vitamin D Safety

Serum concentration of 25(OH)D up to 100 ng/ml is regarded safe in the general population of children and adults, although in preterm neonates, being a specific group, an increased risk of hypercalcemia has been reported at the 25(OH)D values >80 ng/ml (127). No evidence exists until now that these values may be exceeded when appropriate doses of vitamin D are used. In fact, symptoms of vitamin D toxicity are observed very rarely. They are connected with hypercalcemia and hypercalciuria and may occur when vitamin D intake is uncontrolled and excessive, resulting in concentrations of 25(OH)D above 150–200 ng/ml (42, 128). Exceptional conditions comprise individuals with vitamin D hypersensitivity, and also with idiopathic infantile hypercalcemia (IIH) (24, 26), Williams-Beuren syndrome (129), granulomatous diseases (130) and some lymphomas. Vitamin D hypersensitivity may result from impaired catabolism of calcidiol and calcitriol or an excessive, uncontrolled by the feedback, synthesis of calcitriol (local or systemic) (24). The relationship between *CYP24A1* gene mutation and autosomal recessive IIH has been proven (25). It was also shown that subsequently discovered mutations of *CYP24A1* caused loss of function of 24-hydroxylase and clinically manifested as late as in adulthood (131, 132). Hypervitaminosis D may also result from an excessive synthesis of 1,25(OH)_2_D in the case of mutation of *SLC34A1* gene, coding renal sodium-phosphate co-transporter (NaPi-IIA) (26). It is known that in an endocrine (renal) pathway, processes of calcitriol synthesis involving CYP27B1, and its degradation involving CYP24A1, are also regulated by FGF-23. Disorders of phosphorus homeostasis as a result of *SLC34A1* mutation lead to the decrease in FGF-23 activity; FGF-23 in physiological conditions limits activity of 1α-hydroxylase (CYP27B1) and stimulates 24-hydroxylase activity (CYP24A1). A decreased activity of FGF-23 due to *SLC34A1* mutation and subsequent hyperphosphaturia and hypophosphatemia, indirectly stimulate synthesis of 1,25(OH)_2_D that may produce hypercalcemia, hypercalciuria and nephrocalcinosis (133). In case of the diagnosed vitamin D hypersensitivity while supplementing vitamin D deficiency, it is suggested to maintain 25(OH)D concentrations within lower ranges, i.e., 20–25 ng/ml rather than within ranges regarded as optimal, i.e., 30–50 ng/ml (134).

It should be emphasized that at the general population level, vitamin D supplementation with use of daily doses that are recommended for a given age and body mass is safe and reasonable, whereas the incidence of vitamin D hypersensitivity seems to be low or at least it should be precisely investigated. Additionally, upper tolerable limits (UL) have been determined for the general healthy population in order to limit uncontrolled use of vitamin D. Upper tolerable limits should not be confused with recommended doses during well-controlled treatment of vitamin D deficiency. UL are considered safe for the healthy population and are commonly accepted worldwide by international scientific societies [e.g., Institutes of Medicine (IOM, USA) (135), The Endocrine Society (USA) (136) and European Food Safety Authority (European Union) (137)]. In the general population at the neonatal and infantile periods, the UL value equals to 1,000 U/l, in the period of 1–10 years of age—2,000 IU, and from 11 to 18 years of age and in adults—4,000 IU, respectively.

In cases of fully symptomatic vitamin D intoxication, resulting from overdose, the general therapeutic management includes hydration with normal saline followed by loop diuretics and the use of glucocorticoids, bisphosphonates, calcitonin or ketoconazole is often considered as the second-line treatment. Effective therapy is provided also by the anticonvulsant use. Anticonvulsants/antiepileptic drugs are known as potent inductors of cytochrome P450 activity and particularly its isoform CYP3A4. Induction of this enzyme localized in the liver and intestines, contributes to an increase of metabolic clearance of essential vitamin D metabolites, such as 25(OH)D and 1,25(OH)_2_D (138). Polar products of vitamin D, formed in extra hydroxylation pathways, are then quickly excreted from the body (138, 139). When vitamin D status is normal, abovementioned processes result in vitamin D deficiency and mineral disturbances (138–140). Based on the above phenomenon, there have been two successful published attempts of removal of vitamin D excess in neonates (7 and 1.5 months old), casualties of its erroneous overdose (128, 141). In both cases, apart from other modes of treatment, anticonvulsants were administered (phenytoin 5 mg/kg/day for 17 days, phenobarbital 5 mg/kg/day for 133 days in the first case, and phenobarbital 3 mg/kg/day for 4 months in the other case). In both cases, at the end of treatment period, a decrease of 25(OH)D concentrations from approximately 400 to 40 ng/ml and from 160 ng/ml to normal ranges, respectively, was found. An attempt to withdraw phenobarbital (after 44 for 14 days) caused recurrence of intoxication symptoms (128). Eventually, in both cases monitored parameters of mineral metabolism returned to normal values.

### Terminology

The Panel proposed to systematize the terms and nomenclature used in everyday medical practice in Poland. According to panelists’ opinion, it is not justified to use the term “hypovitaminosis D” solely on the basis of 25(OH)D concentration value within the range reflecting vitamin D deficiency. It was observed that clinical symptoms might occur or might not, both at the higher (deficiency) as well as at lower (severe deficiency) ranges of 25(OH)D concentration (<10–20 and 0–10 ng/ml, respectively). This phenomenon may be related to individual sensitivity to vitamin D deficiency state, duration of vitamin D deficiency as well as to the status of mineral metabolism, including calcium intake (8). The terms “symptomatic vitamin deficiency” or “hypovitaminosis D” and “non-symptomatic (subclinical) vitamin D deficiency” should be used depending on the presence or the absence of clinical, biochemical or/and radiological signs. The terms should not be limited only to the recent vitamin D status [25(OH)D concentration], although clinical symptoms are usually observed and may develop along with a decreasing 25(OH)D concentration. Therefore, clinically overt and “symptomatic vitamin D deficiency” or “hypovitaminosis D”, is a state when clinical symptoms coexist with low 25(OH)D concentration value. “Symptomatic hypervitaminosis D” or “vitamin D intoxication” is recognized by markedly elevated 25(OH)D concentration (usually >150 ng/ml) that coincides with normal or slightly increased 1,25(OH)_2_D, hypercalcemia, hypercalciuria and suppressed PTH. The clinical manifestations of vitamin D intoxication are related to hypercalcemia and include: fatigue, weakness, confusion, difficulty in concentration, drowsiness, apathy, vomiting, constipation, polyuria, polydipsia, abnormalities in electrocardiogram (reduced Q-T interval) and others.

## Review of Recommendations

In available literature, the most commonly quoted and discussed position papers are the guidelines elaborated by IOM in 2010 (135), and the practice guidelines issued by the Endocrine Society in 2011 (136).

Based on the evidence available at the time, the IOM focused on calcium and phosphorus metabolism, including benefits limited to bone tissue. As a result, the target for vitamin D supplementation in the general population, recognized by the IOM, was to obtain 25(OH)D concentration of >20 ng/ml (135). In response to the IOM proposals, the recommendations of the Endocrine Society included general healthy population and populations with chronic conditions; furthermore, both the classic and pleiotropic action of vitamin D were incorporated in the integrated guideline. The Endocrine Society’s minimal target value of 25(OH)D concentration was set on 30 ng/ml, and the values of <30 and <20 ng/ml were labeled as insufficient or deficient, respectively (136).

Guidelines concerning the optimal 25(OH)D concentrations and vitamin D supplementation vary across European countries: Scandinavian countries (Denmark, Finland, Iceland, Norway and Sweden) established the target 25(OH)D concentration of ≥20 ng/ml (142), and a similar threshold concept was accepted in Germany, Austria and Switzerland (3).

The recommendations prepared for Central Europe were the closest to the position statement of the Endocrine Society (9). The background in establishing those guidelines were documents by the European Food Safety Authority, published in 2012, and the global discussion of the scientific body over validity and adaptation of the proposals by the IOM and the Endocrine Society. The Guidelines for Central Europe set down the 25(OH)D concentrations of 30–50 ng/ml as optimal in relation to all potential health benefits (9).

Assuming that the aim of vitamin D supplementation is to achieve and to maintain the optimal concentrations of 25(OH)D, as a substrate for renal and extrarenal 1α-hydroxylation (CYP27B1), and in consequence—synthesis of calcitriol, recommendations including endocrine, paracrine and autocrine effects of 1,25(OH)_2_D seem to reflect a holistic view on vitamin D deficiency and human health. The maintenance of recommended optimal 25(OH)D concentrations (>30–50 ng/ml) is reinforced by results of numerous cross-sectional and epidemiological studies, as well as several prospective trials, showing safety of such concentrations, not causing hypercalcemia or hypercalciuria. Another argument supporting 25(OH)D concentration of >30–50 ng/ml as the optimal, involves kinetics of 25-hydroxylase that showed 50% of its activity at the concentration of 40 ng/ml (143, 144). An important evidence was also reported by Priemel et al., who performed histomorphometric analysis of iliac crest bone biopsies in 675 subjects and revealed the osteomalacia lesions in 26% individuals, including 21% of the examined with 25(OH)D concentrations within a range of 21–29 ng/ml (145). Furthermore, osteomalacia signs were not observed in investigated bone biopsies of cases with 25(OH)D concentrations of >30 ng/ml (145). Moreover, studies carried out in pregnant women showed convincing evidence of health benefits for both woman and child that were associated with vitamin D supplementation and with achieved and maintained 25(OH)D concentrations close to 40 ng/ml (146–148).

Global guidelines published in 2016 considered 25(OH)D concentrations of >20 ng/ml as optimal (8). The supplementation regimen in almost all age groups included significantly lower vitamin D doses as compared to those recommended by the Endocrine Society (136) and for Central Europe (9). As a result, also in Poland, practitioners were forced to choose between the global and local recommendations. It should be emphasized, however, that global recommendations consider supplementation only in the context of prevention and treatment of nutritional rickets, and do not refer to other, widely evidenced health benefits related to vitamin D action, as it was pointed out by the authors of that document (8). Interestingly, the vitamin D doses recommended in the global consensus for the management of vitamin D deficiency confirmed by laboratory assays are very similar to the Central European recommendations. In neonates they are even higher (Central European recommendations—1,000 IU/day and global recommendations—2,000 IU/day; Table 4). The global recommendation of using a single loading dose of vitamin D (from 50,000 to 300,000 IU at a time) in treating deficiency in subjects older than 3 months of age, is disputable. A question arises whether it is a return to a historic recommendation of therapy based on a single mega-dose? Absolutely it is not. Loading doses should be justified only in particular situations, when everyday regular supplementation of vitamin D is not possible because of socioeconomic reasons or limitations of the health care system and infrastructure facilitating distribution of vitamin D supplements. In case of loading doses, the risk of hypercalcemia should be carefully taken into account, as it was elsewhere found in 6.5% of children treated with single high doses of vitamin D (8).

**Table 4:** Comparison of recommendations of calcium and vitamin D supplementation for Poland (10), for the Central Europe 2013 (9) and global recommendations of prevention and treatment of nutritional rickets 2016 (8).

	Recommendations for Poland 2009	Recommendations for Central Europe 2013	Global recommendations 2016

	Definition of vitamin D supply based on 25(OH)D concentration in the serum (1 ng/ml = 2.5 nmol/l)		
Optimal concentration (sufficiency)	Children and adolescents: 20–60 ng/ml Adults and seniors: 30–80 ng/ml	>30–50 ng/ml	>20 ng/ml
Suboptimal concentration (insufficiency)	Not defined	>20–30 ng/ml	12–20 ng/ml
Deficiency	<10 ng/ml	0–20 ng/ml	<12 ng/ml
Toxic concentration (toxicity)	Not defined	>100 ng/ml	>100 ng/ml

	**Recommended doses of vitamin D—supplementation (40 IU = 1 µg)**		

0–6 months	400 IU/day	400 IU/day	400 IU/day
6–12 months	400 IU/day	400–600 IU/day	400 IU/day
2–18 years	400 IU/day	600–1,000 IU/day	600 IU/day
>18 years	800–1,000 IU/day	800–2,000 IU/day	600 IU/day
Pregnancy and lactation	800–1,000 IU/day	1,500–2,000 IU/day	600 IU/day

	**Recommended doses of vitamin D—treatment of the deficiency (40 IU = 1 µg)**		

<1 month	1,000 IU/day	1,000 IU/day	–
<3 months	–	–	2,000 IU/day
1–12 months	1,000–3,000 IU/day	1,000–3,000 IU/day	–
3–12 months	–	–	2,000 IU/day
2–19 years	up to 5,000 IU/day	3,000–5,000 IU/day	–
2–12 years	–	–	3,000–6,000 IU/day
>19 years	up to 7,000 IU/day	7,000–10,000 IU/day	–
>12 years	–	–	6,000 IU/day

	**Single loading doses of vitamin D for the management of the deficiency (40 IU = 1 µg)**		

<3 months	Not recommended	Not recommended	Not recommended
3–12 months	Not recommended	Not recommended	50,000 IU/3 months
2–12 years	Not recommended	Not recommended	150,000 IU/3 months
>12 years	Not recommended	Not recommended	300,000 IU/3 months

	**Recommended calcium (elementary) doses**		

0–6 months	300 mg/day	–	200 mg/day
6–12 months	400 mg/day	–	260 mg/day
1–3 years	500 mg/day	–	>500 mg/day
4–6 years	700 mg/day	–	
7–9 years	800 mg/day	–	
10–18 years	1,300 mg/day	–	
19–50 years	1,000 mg/day	–	
>50 years	1,300 mg/day	–	
Pregnancy and lactation			
<19 years	1,300 mg/day	–	
>19 years	1,000 mg/day	–	

In the global consensus, additional attention is paid to relatively low recommended dietary calcium intake, considered as sufficient in preventing nutritional rickets. In children, the following daily calcium intake was recommended: up to 6 months of age—200 mg, 6–12 months—260 mg and after 12 months of age—500 mg. The recommendations for Central Europe (2013) did not arise an issue of calcium intake, however, this was included in Polish recommendations published in 2009 (10). The doses recommended then were definitely higher, particularly in older age groups and increased with age, in the range from 500 to 1,300 mg/day (Table 4). The American Institute of Medicine enforces similar recommendations, including calcium intake of 700–1,300 mg/day for the population aged 1–18 years, depending on a child’s age (135).

## Discussion

Recommendations concerning vitamin D supplementation have been changing over the years and have followed the most recent scientific developments and clinical observations. However, even current doses recommended by scientific societies differ from each other significantly and vary from 200 to 2,000 IU/day (149). This results mainly from discrepancies concerning minimal target 25(OH)D concentration, which was defined by ranges between 10 and 40 ng/ml, depending on how different expert groups perceived vitamin D action (135–137). Most endocrine societies, including the Endocrine Society (USA), and also some dealing with bone health, such as the International Osteoporosis Foundation, reckon the 25(OH)D concentration above 30 ng/ml as that required to achieve health benefits. This value was also determined as a lower range of the optimal 25(OH)D concentration in 2013 Central European recommendations and is now maintained in the present recommendations for Poland (9, 134–137, 150).

The Expert Panel decided on an update of the recommended daily doses for the general population and for the groups at the increased risk of vitamin D deficiency that have been in operation in Poland since 2013. The Panel has decided to add additional target groups for vitamin D supplementation, including adolescents aged 11–18 years, older seniors aged >75 years, and also to modify previous Central European guidelines for the preterm babies.

Indisputably, individuals aged 11–18 years are among the groups of increased risk of vitamin D deficiency, however due to rapid and significant weight gain, an acceleration of skeletal growth, rapid bone turnover and modeling, redistribution of muscle-fat compartments and the other biological and behavioral aspects of pubertal transition, a too low supply of vitamin D during adolescence is of concern. Further, during these critical time frames of development, the risk of vitamin D deficiency and related adverse health outcomes may be exacerbated by sedentary behavior and time spent indoor, dietary habits and even use of restrictive diets. These numerous risk factors for vitamin D deficiency taken together pointed to this group as a target group of special concern and highlighted a need to increase a recommended vitamin D daily dose range to 800–2,000 IU, depending on body weight and season of the year. The British RCT study comprising a group of 110 children and adolescents with normal body weight, aged 14–18 years, evaluated efficacy of vitamin D supplementation at doses of 0, 400 and 800 IU/day, applied in the period between October and March (20 weeks), in order to determine distribution of nutritional requirements to maintain 25(OH)D concentrations ranging from >10 and >20 ng/ml. Data analysis showed that in the examined group of Caucasian children the maintenance of 25(OH)D concentration >10 and >20 ng/ml (in 97.5% of the examined) required a vitamin D supplementation at doses of 400 and 1,200 IU/day, respectively. Interestingly, none of the participants reached the 25(OH)D concentration of 40 ng/ml (151). The RCT of 96 children and adolescents, aged 8–14 years, carried out in the USA (Pittsburg) found that maintenance of 25(OH)D concentrations >20 ng/ml in the period from October to April in 90% of the examined group required vitamin D supplementation at a dose of 1,543 IU/day, whereas an estimated dose of 2,098 IU/day appeared necessary to provide maintenance of this concentration in 97.5% of studied individuals (152). In another RCT, comparing efficacy of vitamin D supplementation applied for 6 months at doses of 600, 1,000 and 2,000 IU/day in the group of 685 school-aged children, the best effects of the supplementation, as expressed by 25(OH)D concentrations of ≥30 ng/ml, were revealed in the 2,000 IU/day group. In this group 25(OH)D concentration of ≥30 ng/ml was obtained in 60% of children already after the 3 months of trial and the use of 2,000 IU/day resulted in the mean 25(OH)D concentration of 33.1 ng/ml (153). The recent study of 1007 Polish children (6), hospitalized due to symptoms of skeletal disorders, revealed that vitamin D deficiency, including a severe vitamin D deficiency (<10 ng/ml), was noted more frequently at the pubertal period and at adolescence as compared to childhood and the prepubertal children, despite the availability of national guidelines.

In the eldest seniors, aged >75 years, according to the Panel opinion, vitamin D should be supplemented throughout the year at doses of 2,000–4,000 IU/day, depending on body weight. The recommended dosing range for the eldest seniors up to 4,000 IU/day was considered by panelists as effective enough to achieve the target 25(OH)D concentration of >30–50 ng/ml in at least 90% of the elderly in Poland. The group of the eldest seniors is another target group at increased risk of vitamin D deficiency, as well as falls and fragility fractures. Available RCT studies and meta-analyses evidenced that 25(OH)D concentrations ranging >24–50 ng/ml, as a result of vitamin D supplementation of seniors and the eldest seniors, were associated with a significant decrease of risk of falls (by 19%) (154), a significant decrease of risk of proximal femoral fractures (by 37%) (155) and significantly decreased risk of other fractures (by 31%), compared to controls. Although most studies reviewed recommended supplemental doses >800 IU/day, still about half of the seniors and the eldest seniors supplemented with vitamin D did not reach 25(OH)D concentrations considered as optimal. Therefore, after numerous discussions, the Expert Panel recommended a full eradication of vitamin D deficiency, using doses 2,000–4,000 IU/day in order to achieve and maintain the optimal 25(OH)D concentration and also to provide the eldest seniors with potential benefits resulting from pleiotropic vitamin D action. The above recommendation is well-matched to the American Geriatric Society guidelines (156).

The Expert Panel, basing on the review of the literature and RCT studies, has decided to modify the Central European recommendations for preterm babies. RCT studies published during the last 5 years revealed advantages of vitamin D supplementation at doses of 800–1,000 IU/day in neonates born at ≤32 weeks of gestation and in neonates born with very low birth weight (<1,500 g) (157–159). In the study comparing effects of vitamin D supplementation (1,000 vs 800 vs 400 IU/day), the percentage of the preterms with vitamin D deficiency at 36 weeks of the postmenstrual age was 2.5, 9.8 and 22.5%, respectively (159). In a group of more preterm babies (born at ≤28 weeks of gestation), after 4 weeks of vitamin D supplementation (800 vs 200 IU/day vs placebo), the percentage of the preterms with vitamin D deficiency was 0, 16 and 41%, respectively (158). In the subgroup supplemented with vitamin D dose of 800 IU/day, the majority of investigated cases reached 25(OH)D concentrations >60 ng/ml, despite that as high as 67% preterms presented vitamin D deficiency at birth. An observational study of 66 preterm neonates (mean birth weight 970 g, 27 weeks of gestation) showed that vitamin D supplementation at a dose of 800 IU/day was effective to reduce prevalence of severe vitamin D deficiency, evaluated at 36 weeks of the postmenstrual age, from 41 to 0%, as well as to improve prevalence rate of 25(OH)D concentrations >30 ng/ml from 10 to 72% (160). Unfortunately, the problem of vitamin D deficiency in the preterm neonates is also common in Poland (161). The risk of vitamin D deficiency at birth rises along with the shortening of pregnancy duration and the risk of preterm delivery increases with severity of vitamin D deficiency in pregnant women (146–148). The updated vitamin D supplementation doses seem effective for the quick improvement of vitamin D status of preterm neonates, however, after a one month of vitamin D supplementation, according to panelists guidelines for preterms, it is recommended to evaluate 25(OH)D concentration and if necessary modify the dosage. Because of the concern about adverse effects and risk of overdosing, studies with lower vitamin D doses (200 IU/day) were also conducted in the preterms born at ≤32 weeks of gestation. At the 36 week of the postmenstrual age, vitamin D supplementation at a dose of 200 IU/day appeared not fully effective and vitamin D deficiency was noted in up to 40% of cases born at <28 weeks of gestation and 30% of cases born at 28–32 weeks of gestation (162). It seems that in more mature preterm neonates (with relatively lower risk of the severe vitamin D deficiency), vitamin D supplementation at a dose of 400 IU/day, that is also recommended for in-term born neonates, should provide adequate vitamin D supply (163).

The Expert Panel is of the opinion that population-based 25(OH)D concentration screening is not justified, however recognizes strong indications for 25(OH)D concentration assessment in an increasing number of clinical conditions in order to optimize the course and to minimize complications of the underlying disease (Table 1). The Panel shares the position statements of the Endocrine Society that the vitamin D supplementation in groups at risk of vitamin D deficiency that need special concern, including women planning pregnancy, pregnant and lactating women and the preterm babies (<32 weeks gestation) should be provided and followed under the control of 25(OH)D concentration and its changes (136). Some available reports pointed on vitamin D supplementation doses of 4,000–6,400 IU/day as safe, effective and beneficial for pregnant and lactating women as well as for offspring (146–148). In contrast, studies performed in Poland and Canada (Calgary, 51°N) evidenced a low effectiveness of vitamin D supplementation at doses 600–800 IU/day for beneficial pregnancy outcomes (16, 164). Considering the safety of vitamin D supplementation during pregnancy (to a lesser extent during lactation) and a high probability of use of multicomponent preparations that usually contain 200 IU vitamin D per serving, the Expert Panel recommends the dose of 2,000 IU/day for the general population of pregnant and lactating women with unknown 25(OH)D concentration. Some pregnant women may require higher doses of vitamin D to achieve optimal 25(OH)D concentration, however, vitamin D supplementation using doses higher than 2,000 IU/day should be carried out based on initial 25(OH)D concentration and its change. The Panel underlines that vitamin D deficiency during pregnancy is associated with a significantly higher risk of preterm delivery and preeclampsia and is considered as a risk factor for low birth weight and bacterial vaginosis. Correction of vitamin D deficiency by regular vitamin D supplementation during pregnancy, starting as early as possible (preferably at the pre-conception stage), may markedly reduce the risk of abovementioned complications and therefore is highly recommended (146–148, 165, 166).

Keeping in mind reports on a group of patients in the Polish population who are genetically predisposed to symptomatic hypercalcemia (carrying *CYP24A1* or *SLC34A1* gene mutations, resulting in decreased catabolism or excessive formation of an active form of vitamin D, respectively) (26), the Expert Panel suggests to consider a directed medical history investigation, anteceding vitamin D supplementation with vitamin D doses higher than recommended for the general population, in order to minimize the risk of adverse events in a individuals with vitamin D hypersensitivity. It was estimated that at least a thousand cases predisposed to symptomatic hypercalcemia live in Poland and the prevalence may be as high as 1:33.000 births (26). If there is a diagnosis of hypercalcemia, hypercalciuria, nephrolithiasis, nephrocalcinosis, *CYP24A1* or *SLC34A1* gene mutations or other form of vitamin D hypersensitivity in a patient or his/her family members, the supplementation should be carried out individually, and controlled by parameters of calcium-phosphate metabolism, particularly calcemia, PTH, calciuria, 25(OH)D and 1,25(OH)_2_D.

In the context of therapeutic dosing of vitamin D, the Expert Panel is of the opinion that single loading doses of vitamin D provided at the 3-month intervals should not be recommended in Poland. An approach for the loading doses use was proposed in the 2016, by global consensus focused on prevention and therapy of nutritional rickets, as an alternative therapeutic procedure for patients suffering from nutritional rickets, exclusively if the regular daily vitamin D supplementation is not possible for various reasons (8). Taking into account a higher risk of hypercalcemia as a result of loading doses (8) and previous Polish and European experience with loading doses (42), as well as a relatively easy, permanent access to vitamin D supplements and health care in Poland, an implementation of very high doses would not be justified.

Recently, a regimen of a single dose of 30,000 IU of vitamin D_3_ available on prescription, has been promoted in Poland, with an indication for administration once a month in adults, the elderly and adolescents older than 12 years of age. The standpoint of the Expert Panel is that vitamin D intake at a dose of 30,000 IU, regardless the regimen [once a month according to summary of product characteristics (SPC) or more often according to some positions (167)], is considered neither as appropriate nor as safe management, if a prior assessment of 25(OH)D concentration and the risk factors for vitamin D hypersensitivity were not investigated. In the aspect of prevention of vitamin D deficiency in the general population (considered as healthy) or even in the groups of risk of deficiency, vitamin D supplementation at a cumulative dose equivalent to 15 or 30 daily doses (2,000 or 1,000 IU/day, respectively) rises concerns about safety. In the most extreme regimen of supplementation with use of cumulative dose recently promoted in Poland (30,000 IU), for example in older obese adolescents and obese adults, the recommendation of doses constituting two to three times the dose recommended for peers with normal body weight (i.e., 60,000–90,000 IU, respectively) even twice a month according to some reports (167), should be considered unwarranted and very risky. Panel is of the opinion that vitamin D supplementation at a single dose of 30,000 IU, diverging from the SPC (once a month) may be unfavorable even as an adjunct to osteoporosis treatment. In a RCT of 200 subjects aged >75 years an increased risk of falls as a result of use of 24,000 IU with 300 µg of calcifediol once a month, as well as an increased risk of falls as a result of 60,000 IU once a month were both evidenced (168).

The Expert Panel in the updated Polish recommendations as well as others [the previous Endocrine Society (136) and Central European (9) guidelines] do acknowledge abnormal body weight as a significant variable affecting vitamin D status and recommend obese persons from general population a doubled daily dose of vitamin D. A weak but significant negative correlations were shown between 25(OH)D concentrations and body weight as well as BMI (kg/m^2^) in Poland (7). However, vitamin D deficiency is more often noted in obese persons, irrespective of age (169). Our guidelines recommend a two times higher daily dose for obese persons in relation to normal body weight counterparts. Further, age- and body weight related ranges of vitamin D for daily dosing, proposed by experts for use in general population, most likely will help to deal also with underweight cases. Our approach is consistent with the Endocrine Society’s recommendations (136) and is supported by results of large surveys that estimated two to three times higher vitamin D daily dose for obese subjects, 1.5 times for overweight, and pointed that underweight persons may need lower vitamin D doses to achieve target 25(OH)D concentration when compared to individuals with normal body weight (170–172).

**Figure 1:**
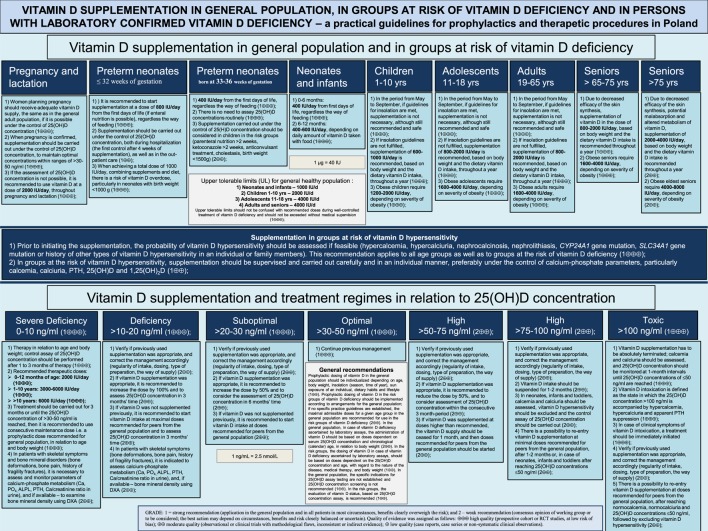
The chart summarizing practical guidelines.

The Expert Panel recommends that vitamin D supplementation in individuals with an assayed 25(OH)D concentration should be based on the vitamin D status diagnosed according to recommended concentration ranges and should consider previous prophylactic management. Above statement relates to common observation that guidelines for vitamin D supplementation are not implemented or are not carried out properly and the fundamental problem is non-compliance (5, 6). In individuals declaring supplementary vitamin D intake with revealed abnormal 25(OH)D concentration value (low, high, too high, too low, etc.) the first line of management should be based on evaluation of regularity of vitamin D use, the dosage, a choice of preparation and the way of administration (with or without fat-containing products—depending on the preparation). A simple correction of management of vitamin D deficiency usually is sufficient enough. However, if the vitamin D supplementation was compliant to recommended but a response was not satisfying, expressed as 25(OH)D concentration still below optimal value range, it is recommended to increase a daily dose by 50–100% or to introduce therapeutic doses—depending on a severity of vitamin D deficiency. If vitamin D supplementation was not so far implemented, it should be started immediately, including the use of therapeutic doses in individuals showing severe deficiency (25(OH)D <10 ng/ml) (Figure 1). The 25(OH)D follow-up and the range of additional investigations should depend on a severity of vitamin D deficiency.

The Expert Panel emphasizes significance of appropriate dietary calcium intake during the course of vitamin D supplementation and treatment of vitamin D deficiency. If dietary sources are considered as not effective, additional pharmacological supplementation with calcium salts preparations is recommended, preferably in a few divided daily doses due to higher absorption rate and the lower risk of periodic hypercalciuria. At the current state of knowledge, it is recommended to maintain existing calcium intake guidelines for Polish population, depending on the age (10).

## Summary

Vitamin D deficiency is an important public health problem in Poland that without appropriate preventive actions may escalate as a result of the on-going changes of life style, unfavorable nutritional habits as well as limited vitamin D supply from natural sources (both dietary and UVB) (4–7). It is necessary to introduce and pursue recommendations in its updated form concerning recent perspective on the prevention and treatment of vitamin D deficiency in all age groups. The synopsis of the guidelines is shown in Figure 1. This task should be a priority for doctors of all specialties, primarily for general practitioners, as well as people shaping health policies in Poland. It is essential particularly in the context of present knowledge, which provides evidence not only of calcemic action of vitamin D, but also of its pleiotropic effects. The list of classic and non-classic—pleiotropic action of vitamin D and associated health benefits becomes longer and longer. The optimal 25(OH)D concentrations for different endocrine, autocrine and paracrine pathways are indicated as an essential factor in preventing osteoporosis and falls, rickets and osteomalacia, as well as autoimmune diseases, including multiple sclerosis, diabetes type 1, systemic lupus erythematosus, infectious diseases, including tuberculosis and influenza, cardiovascular diseases, neurocognitive disorders, including Alzheimer disease, autism, pregnancy complications, diabetes type 2, as well as decrease of incidence rate and improvement of survival rate and quality of life in malignancy and overall mortality. Despite discussions on causality between vitamin D deficiency and a given disease or a risk of its development, and also in a view of the magnitude of the problem of vitamin D deficiency in the general population and in patients, the Expert Panel recommends implementation of updated guidelines dedicated to prevention and treatment of vitamin D deficiency to a routine everyday practice of physicians and clinical dieticians.

## Author Contributions

All authors contributed to the preparation of the guidelines, all participated in the data collection, drafting, writing and editing the manuscript. MWa is the National Consultant in Pediatric Endocrinology and Diabetes; President of the Polish Society of Pediatric Endocrinology and Diabetes. MB-K is the President of the Polish Society of Neonatology. DCH-S is the Chairwoman of the Section of Bone Metabolic Diseases in Children and Adolescents at the Polish Pediatric Society. EH is the National Consultant in Neonatology. TJ is the National Consultant in Pediatrics. JKs is the President of the Polish Society for Clinical Nutrition of Children. AL is the National Consultant in Endocrinology. JP-P is the President of the Polish Pediatric Society. MR is the President of the Polish Society of Endocrinology. MWi is the National Consultant in Perinatology; President of the Polish Society of Gynecologists and Obstetricians. DZ is the National Consultant in Pediatric Nephrology. PP is the President of the European Vitamin D Association—EVIDAS.

## Conflict of Interest Statement

The authors declare that the research was conducted in the absence of any commercial or financial relationships that could be construed as a potential conflict of interest. The handling Editor is currently co-organizing a Research Topic with the authors PP and JKo and confirms the absence of any other collaboration.

## Abbreviations

1,25(OH)_2_D, 1,25-dihydroxycholecalciferol; UVB, ultraviolet radiation B; DBP, vitamin D-binding protein; MED, minimal erythemal dose; 7-DHC, 7-dehydrocholesterol; D3, vitamin D3, cholecalciferol; D2, vitamin D2, ergocalciferol; DXA, dual-energy X-ray absorptiometry; 25(OH)D, 25-hydroxyvitamin D, calcidiol, calcifediol; FGF-23, fibroblast growth factor 23, phosphatonin-23; RANK, receptor activator of nuclear factor NF-κB; RANKL, RANK ligand; RCT, randomized controlled trials; PTH, parathormone; VDR, vitamin D receptor; ALPL, alkaline phosphatase activity; PO_4_, phosphate; Ca, calcium.
